# Proper connectivity of *Drosophila* motion detector neurons requires Atonal function in progenitor cells

**DOI:** 10.1186/1749-8104-9-4

**Published:** 2014-02-26

**Authors:** Carlos Oliva, Ching-Man Choi, Laura J J Nicolai, Natalia Mora, Natalie De Geest, Bassem A Hassan

**Affiliations:** 1 VIB Center for Biology of Disease, VIB, 3000 Leuven, Belgium; 2 Center for Human Genetics, University of Leuven School of Medicine, 3000 Leuven, Belgium; 3 Program in Molecular and Developmental Genetics, Doctoral School for Biomedical Sciences, University of Leuven School of Medicine, 3000 Leuven, Belgium

**Keywords:** neural progenitor, *Drosophila*, atonal, neurite guidance

## Abstract

**Background:**

Vertebrates and invertebrates obtain visual motion information by channeling moving visual cues perceived by the retina through specific motion sensitive synaptic relays in the brain. In *Drosophila*, the series of synaptic relays forming the optic lobe are known as the lamina, medulla, lobula and lobula plate neuropiles. The fly’s motion detection output neurons, called the T4 and T5 cells, reside in the lobula plate. Adult optic lobe neurons are derived from larval neural progenitors in two proliferating compartments known as the outer and inner proliferation centers (OPC and IPC). Important insight has been gained into molecular mechanisms involved in the development of the lamina and medulla from the OPC, though less is known about the development of the lobula and lobula plate.

**Results:**

Here we show that the proneural gene Atonal is expressed in a subset of IPC progenitors that give rise to the higher order motion detection neurons, T4 and T5, of the lobula plate. We also show that Atonal does not act as a proneural gene in this context. Rather, it is required specifically in IPC neural progenitors to regulate neurite outgrowth in the neuronal progeny.

**Conclusions:**

Our findings reveal that a proneural gene is expressed in progenitors but is required for neurite development of their progeny neurons. This suggests that transcriptional programs initiated specifically in progenitors are necessary for subsequent neuronal morphogenesis.

## Background

In animals, visual information is collected using photoreceptor cells, which send electrical signals to the central nervous system were the light stimulus is processed. *Drosophila* is a particularly well-characterized model for studying genetic mechanisms involved in neuron development and physiology. A fly’s eye is composed of 800 repetitive units called ommatidia, which collect light from the environment. Each ommatidium has eight photoreceptor neurons and twelve accessory cells. The axons of the photoreceptors reach to the optic lobe of the fly brain, where the visual information is further processed [1]. The optic lobe of a fly consists of around 60,000 cells distributed over four neuropiles: the lamina (La), medulla (Me), lobula (Lo) and lobula plate (Lop). These four neuropiles derive from two major larval optic lobe progenitor domains called the outer proliferation center (OPC) and the inner proliferation center (IPC) [2]. The mechanisms that give rise to the neural progenitors and regulate their division have been intensively investigated [2,3]. It has been previously established that the OPC give rises to the lamina and the outer medulla and the IPC to the inner medulla, lobula and lobula plate [4,5]. Many of the neurons that reside in the optic lobe have been characterized by Golgi impregnation [6]. They have characteristically different morphologies; however, the molecular bases that underlie these morphologies have not been established. So far, differentiation of the photoreceptor [1], lamina [7] and medulla [8] has been addressed in great detail. In contrast, specific lineage details are largely unknown for the lobula and lobula plate. For example, it is entirely unclear whether specific precursor subtypes give rise to specific neuronal subtypes, how such lineages develop and what genes regulate their development.

In both flies and mammals, the transition from neural progenitors to neurons is governed to a great extent by highly conserved transcription factors of the basic helix-loop-helix (bHLH) family, which are known as proneural proteins [9-11]. There are two major classes of proneural proteins, called the Achaete-Scute (AS) family and the Atonal (Ato) family, after their founding members. First functionally described for the *Drosophila* peripheral nervous system (PNS), the two types of proteins act as transcriptional activators regulating the commitment of distinct subsets of peripheral epithelial cells to neural fate [12]. Ato has been shown to be expressed in the fly optic lobe during development [10]. However, so far the nature of these cells and the function of Atonal during their development have not been addressed. In this work we show that Ato is expressed in a group of neural progenitors in the IPC that give rise exclusively to T4 and T5 lobula plate neurons, which are motion detection neurons [13]. Furthermore we find that Ato does not act as a proneural gene in this context, but instead it regulates the connectivity of T4/T5 neurons, suggesting that a transcriptional program initiated in progenitors regulates aspects of terminal differentiation in neurons.

## Results

### Ato is expressed in precursors of the inner optic lobe

**Figure 1:**
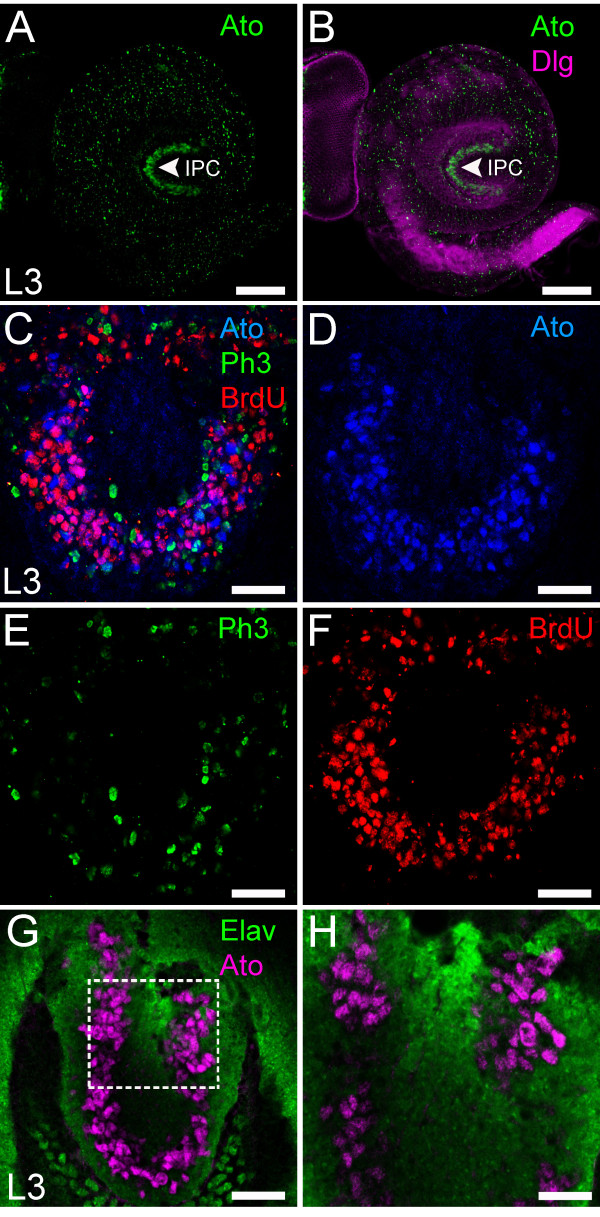
**Ato is expressed in the IPC. (A,B)** Ato is expressed in the IPC (arrow) in L3 (green). Dlg was used to mark all cells (magenta). **(C-F)** Ato^+^ cells are proliferative. The S phase marker BrdU (red in (**C**) and (**F**)) but not the mitosis marker Phospho-Histone-H3 (green in (**C**) and (**E**)) is present in Ato^+^ cells (blue in (**C**) and (**D**)) in the IPC. **(G,H)** Ato is not expressed in neurons in the IPC. Ato (magenta) and Elav (green) show no co-localization. **(H)** High magnification of the region in **(G)** (single section). Scale bars: A,B = 50 μm, C,F = 20 μm, G = 20 μm, H = 8 μm. IPC, inner proliferation center; L3, third instar larvae.

The *Drosophila* larval optic lobe has been successfully used as model to study the regulation of neural differentiation [14-16], but much less is understood about the genetic control during the development of neuronal subtypes. The four neuropiles of the adult *Drosophila* optic lobe are formed from two populations of progenitors visible within the developing brain at the third larval instar stage (L3), the OPC and IPC. The ubiquitous epithelial marker Discs Large (Dlg) can be used to highlight the general architecture of the developing L3 nervous system (Figure 1A,B) including neuronal precursors and neuropiles [4,17]. The highly conserved proneural transcription factor and tumor suppressor gene Ato is required for the proper development of the fly visual system. Ato mutants lack the retina and have severe defects in the optic lobes [18], largely as a non-autonomous result of the loss of retinal neurons [19]. However, in addition to its expression in the retina, previous reports noted the expression of Ato in the larval optic lobe, including expression close to the IPC [10,18]. This suggests that *ato* might play an additional role in the development of the fly visual system.

To gain insight into optic lobe development and the potential function of the Ato proneural protein within it, Ato expression in the developing optic lobes was examined in further detail. The localization of Ato^+^ cells in the IPC (Figure 1B) suggests that they are neuronal precursors rather than neurons. To address this possibility we stained L3 brains treated with a short pulse of bromodeoxyuridine (BrdU) using Ato antibodies in order to label proliferating progenitors (Figure 1C,F). We found that Ato is co-localized with BrdU, indicating that Ato is expressed in a subset of IPC progenitors during the S phase. However, there was little overlap between Ato and the mitotic marker Phospho-Histone-H3 (PH3), suggesting that Ato is rapidly downregulated prior to progenitor cell division. On the other hand, Ato does not co-localize with the neuronal marker Embryonic lethal abnormal vision (Elav) (1G,H), indicating that it is not expressed in neurons. In summary, Ato marks a subset of progenitors in the developing *Drosophila* optic lobes.

**Figure 2:**
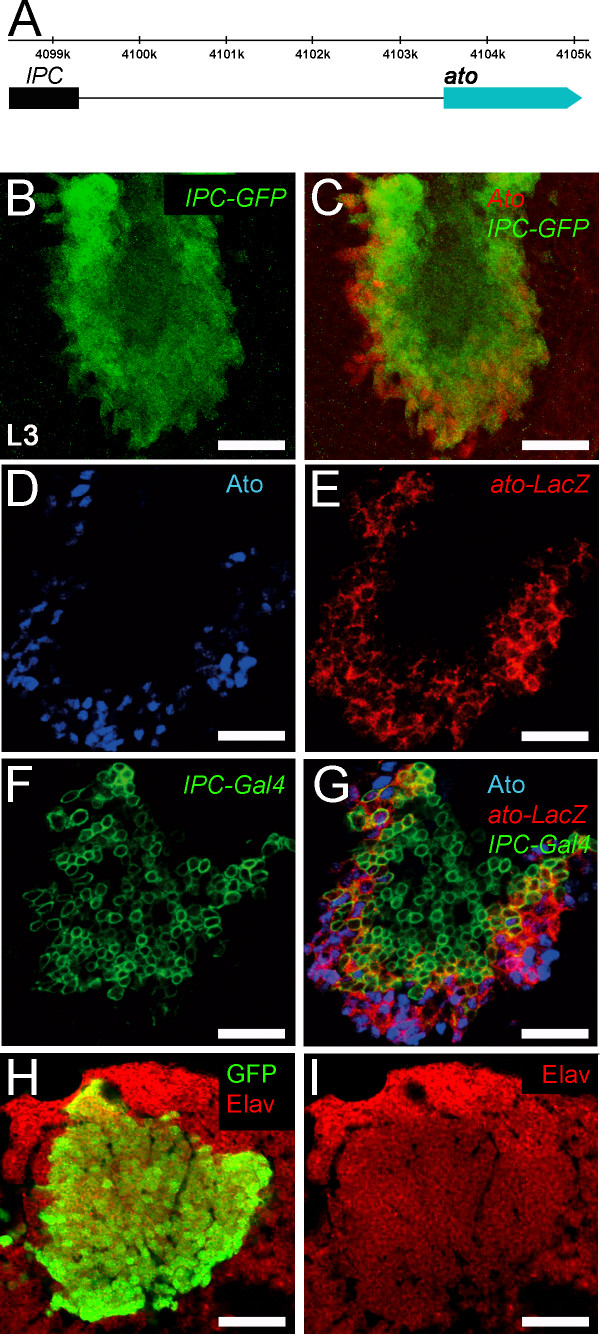
**Ato IPC enhancer characterization. (A)** A region upstream of the promoter of ato between the positions -5046 and -4209 drives expression in the IPC. **(B-I)** Immunostaining of L3 optic lobes using the indicated antibodies. **(B,C)***IPC-GFP* direct fusion **(B)** co-localizes with almost all Ato^+^ cells and persists in the progeny **(C)**. **(D-E)** Ato^+^ cells (**D**, blue) co-localize completely with the *ato-LacZ* reporter (**E**, red). LacZ persists in the immediate progeny. *IPC-Gal4*, UAS-CD8-GFP are delayed. GFP co-localizes with very few Ato^+^ cells **(G)** but it is present in the immediate LacZ^+^ progeny as well as at later stages (green). **(H,I)** GFP^+^ cells are Elav^+^ neurons. Scale bars: B-I = 20 μm, IPC, inner proliferation center.

We sought to identify the lineage of Ato^+^ progenitors and investigate the role of *ato* in inner optic lobe development. We generated an IPC-specific *ato-Gal4* driver line that is not expressed in the retina. We have previously described an *ato-LacZ* reporter line, which mimics *ato* expression in the L3 brain but not in the eye imaginal disc [10]. This *LacZ* reporter is expressed in the same cells as the Ato protein and its expression lasts longer due to the stability of the β-Gal protein. A similar, but slightly shorter version of this enhancer was used first to generate a direct GFP fusion reporter line to ascertain that it labels the same Ato^+^ lineage. The enhancer was then used to generate an *ato-Gal4* driver specific to the IPC (Figure 2A), which henceforth will be referred to as *IPC-Gal4* or the IPC driver. The overlap between Ato, *IPC-GFP*, *ato-LacZ* and *IPC-Gal4* shows that these two enhancers identify the same population of cells (Figure 2B,C,D,E,G). The green fluorescent protein (GFP) is known to be highly stable. Expression of GFP using *IPC-Gal4* marks the derivatives of the Ato^+^ progenitors and it was only present in some precursors, presumably the oldest pool, due to the delay in GFP expression and the stability of GFP (Figure 2F,G, green). During the L3 stage, the progeny of Ato^+^ cells move towards the center of the IPC, where they differentiate into neurons as demonstrated by co-localization of the pan-neuronal markers Elav and GFP (Figure 2H,I). Finally, a LexA knock-in into the Ato locus [20] also co-localizes with *IPC-Gal4*-driven GFP at the border of the cluster, presumably the oldest pool of Ato^+^ progenitors (Additional file 1: Figure S1). In summary, Ato^+^ progenitors in the IPC generate a neuron population resident in the optic lobe.

### Atonal progenitors give rise exclusively to motion detection T4 and T5 neurons

**Figure 3:**
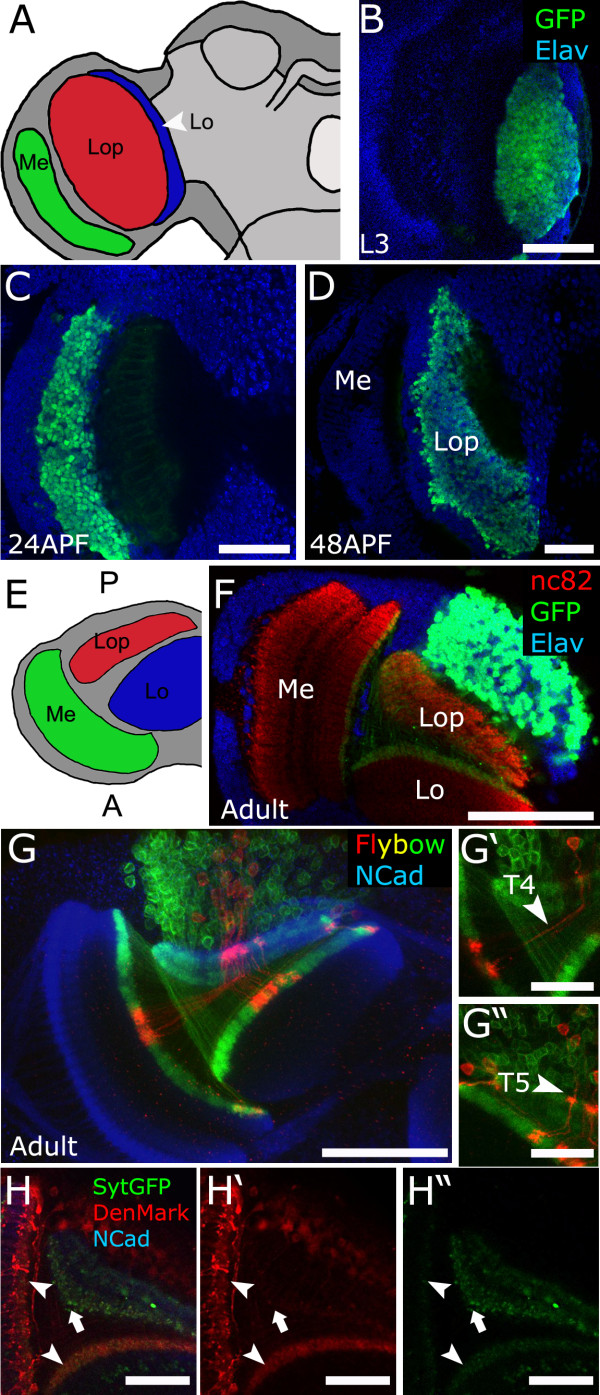
**Ato**^**+ **^**progenitors give rise to T4 and T5 lobula plate neurons. (A)** Components of the fly optic lobe (posterior view). **(B-F)** Animals bearing *IPC-Gal4*, *UAS-nEGFP* were used to visualize the progeny of Ato^+^ cells in different stages of development (green). Elav was used to highlight neurons. **(B)** L3, **(C)** 24hAPF, **(D)** 48hAPF. **(E)** Horizontal view of the brain. **(F)** Immunostaining in adult stage (horizontal section) showing the pattern of T4/T5 neurons. This population of neurons is localized at the rear of the Lop (at the top of the panel). **(G)** Immunostaining of optical horizontal sections of the optic lobe of adult brains of flies bearing transgenes for the flybow technique. Flip-out and inversions events of the flybow 2.0 cassette were visualized with anti-GFP (green) and anti-DsRed (red) antibodies. NCad (blue) was used as general neuropile marker. **(G',G'')** High magnification images of single neurons. Arrowheads indicate single T4 and T5 neurons. **(H,H',H'')** Adult brains (horizontal section) expressing *UAS-Denmark* (red) and *UAS-Synaptotagmin-GFP* (*UAS-SytGFP*, green) driven by *IPC-Gal4*. Denmark is enriched in the proximal Lo and proximal Me (arrowheads) while SytGFP is enriched in the Lop neuropile (arrow). Scale bars: B-D, F = 50 μm, G = 50 μm, G’-G'' = 25 μm, H-H'' = 25 μm. A, anterior; L3, third instar larvae; Lo, lobula; Lop, lobula plate; Me, medulla; P, posterior.

Next, we analyzed the expression of *IPC*-*Gal4* throughout development (Figure 3), to trace the Ato lineage. During pupal development, neurons generated from Ato^+^ precursors become localized in the posterior part of the optic lobe in the Lop. This can also be clearly seen in confocal horizontal sections through an adult fly brain (Figure 3F). Thus Ato^+^ progenitors give rise to a group of neurons that populate the rear of the Lop.

It has been shown that the posterior cortex of the Lop contains three major neuronal subtypes, called T4/T5, the translobula plate (Tlp) and Y. T4 and T5 neurons are characterized by their peculiar looping trajectory. They project through the Lop towards the proximal Me (T4) or proximal Lo (T5) where they form a dendritic arborization. They then turn 180° to project back towards the Lop where they form axonal endings. The other two classes, Tlp and Y, form dendritic arbors in the Lop, and axonal terminations in the Lo (Tlp) and proximal Me (Y) [6]. We sought to determine which specific Lop neurons originate from Ato^+^ progenitors. We used the flybow technique, which is used to mark stochastically clones of cells with different fluorescent proteins [21]. Using this approach with *IPC-Gal4* and examining multi- and single-cell clones, we found only two populations of neurons, one sending projections towards the inner medulla and the other towards the lobula (Figure 3G,G',G''). The morphology observed resembles the T4 and T5 neurons described previously [6] and recently found to be essential components of the motion detection circuit [22,23]. Based on the morphology shown by silver staining, it has been proposed that dendrites of these neurons project towards the Lo (T5) and Me (T4) while their axons project within the Lop. To confirm this, we used subcellular markers to determine which projections correspond to dendrites and axons (Figure 3H,H',H''). Consistent with the Ato^+^ derived neurons being T4 and T5, we find that the dendritic marker DenMark [24] localize in the projections targeting the proximal layers of medulla and lobula, respectively, whereas the presynaptic vesicle marker Syt-GFP labels the projections in the Lop. In summary, Ato^+^ progenitors give rise mostly or exclusively to T4/T5 neurons, although an additional small minority population that escapes clonal labeling cannot be ruled out at this stage.

### *Atonal* does not act as a proneural gene in the optic lobe but is required for neurite guidance

To address the role of *ato* in optic lobe development, we examined the neuronal progeny of Ato^+^ precursors in *ato* mutant animals using two independent mutant alleles: the *ato*^1^ mutant [18] and a knock-in of the *white* gene in the *ato* locus [25]. One copy of *ato* is sufficient to form a phenotypically wild-type optic lobe, therefore *ato/+* animals were used as controls. In the PNS, loss of *ato* results in the complete loss of sensory precursors and thus the sensory lineages derived from them [18,26]. We found that in the IPC, however, Ato is not required to generate optic lobe neurons, since in the absence of *ato*, the Ato^+^ precursors still gave rise to neurons. The pan-neuronal marker Elav remained almost completely co-localized with the GFP^+^ cells as in wild-type L3 brains (Additional file 2: Figure S2). Moreover we observed that the area of the neuronal cluster is comparable between mutant and control animals (Additional file 2: Figure S2C). This indicates that *ato* does not act as a proneural gene in the optic lobe in contrast with its function in PNS progenitors.

**Figure 4:**
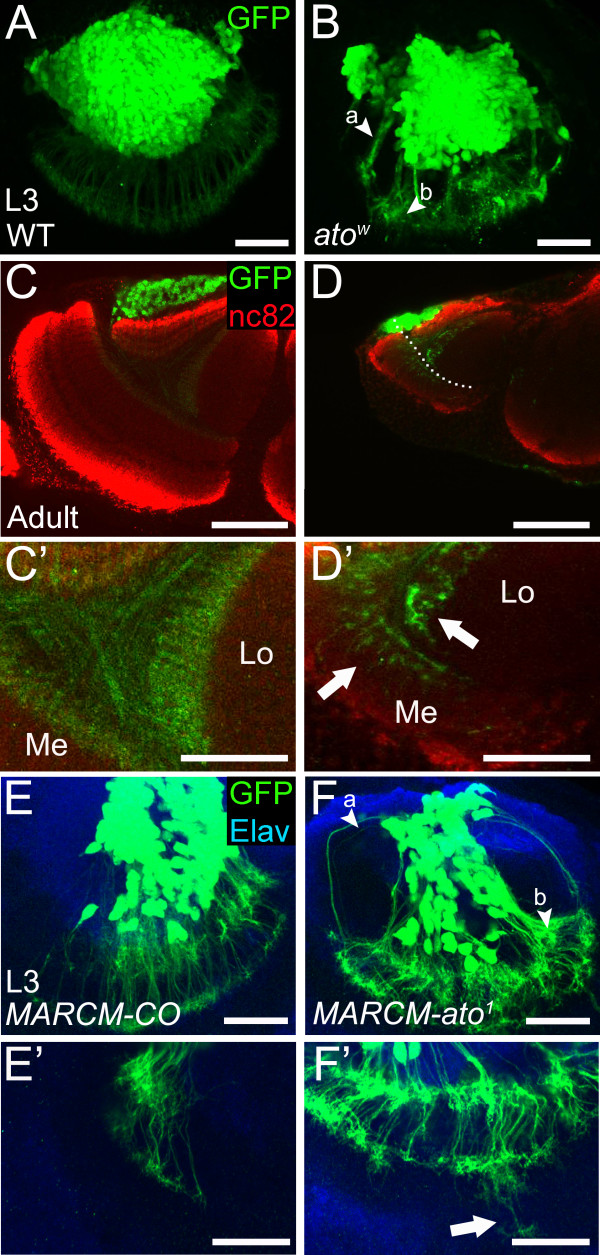
**Ato is required for neurite guidance in progeny neurons. (A-F’)** Neurite guidance defects are observed in *ato* mutant in L3 and adult brains. **(A,B)** Immunostaining of wild-type and *ato*^*w *^L3 cells bearing GFP (green) driven by *IPC-Gal4*, *UAS-nEGFP*. Note misguidance (a) and over fasciculation (b) of the neurites (arrowheads in (**B**)). **(C',D')** Mutant phenotypes in adult animals. **(C,C')** Wild-type cells bearing *IPC-Gal4*, *UAS-nEGFP*. **(C')** Close-up showing the axonal and dendritic projections. **(D,D')***ato*^*w *^mutant. Reduction in Me and Lo complex sizes is clear. **(D')** Close-up showing the axonal and dendritic projections. Some dendrites have growth and fasciculation defects (arrows). Dotted line in **(D)** marks the border between the Lo complex and Me. **(E,F)***ato* mutant phenotypes in MARCM clones. (Arrowhead a: guidance defects; arrowhead b: fasciculation defect) **(E',F')** Close-ups of different samples in the neurite region showing overgrowth defects in mutant MARCM clones (arrow in (**F'**)). Scale bars: A-B = 20 μm, C-D = 50 μm, C'-D' = 25 μm, E,F,E',F' = 20 μm. L3, third instar larvae; Lo, lobula; Me, medulla, WT|, wild type.

To further investigate if Ato is required for the development of T4/T5 development, we examined their projection pattern in L3 and adult stages. In developing T4/T5 neurons, the neurites form a radial arrangement around the cell body cluster (Figure 4A). In contrast, in *ato* mutants neurites lose this organization, showing over fasciculation, guidance and growth defects (Figure 4B, Table 1). We observed a similar phenotype in the optic lobe of adult animals where dendrites have clear fasciculation and overgrowth defects (Figure 4C,D,D'). However, in full *ato* mutants the retina and La were completely absent and the Me and Lop neuropiles were markedly reduced in size, as can be appreciated by comparing wild-type with mutant flies. It has been previously established that the retina is required for neurogenesis in the larval optic lobes [19] and as such optic lobe defects and T4/T5 defects in *ato* mutants may be a consequence of the loss of the retina. To test this, we generated animals in which most of the retina was absent in an otherwise wild-type background by expressing the cell death gene *hid* with the eye-specific Glass multiple reporter (*GMR*) promoter during eye development (Additional file 3: Figure S3A,B,B'). This resulted in essentially the complete loss of the La (Additional file 3: Figure S3B,B'). In addition, the Me appeared reduced in size, while the size and overall organization of the Lo and Lop appeared significantly better. Specifically, the general organization of the T4/T5 neurons was largely unaffected in contrast to the *ato* mutant brains where the T4/T5 neurons were disorganized. This suggests that T4/T5 defects observed in *ato* mutants may be specifically due to loss of Ato in the IPC. To confirm this, we generated MARCM [27] clones using the *ato*^*1*^ allele (Figure 4E-F', Table 1) and analyzed the T4/T5 neurons at the L3 stage. We observed similar phenotypes to the ones seen in the whole mutant, where the neurites over fasciculate and are misguided and there is some overgrowth towards the central brain, further confirming that Atonal is required in the IPC for normal T4/T5 development.

**Table 1:** **Quantification of neurite defects observed in *atonal *** **mutants and MARCM clones**

**Genotype**	**Neurite guidance**	**Neurite growth**	**Fasciculation**	***n***
ato^w^ /+	0/27 (0%)	0/27 (0%)	0/27 (0%)	27
ato^w^/ato^w^	26/26 (100%)	12/26 (46%)	26/26 (100%)	26
CO_MARCM	2/25 (8%)	1/25 (4%)	1/25 (4%)	25
ato^1^_MARCM	16/25 (64%)	12/25 (48%)	9/25 (36%)	25

In summary, Ato does not act as a proneural factor in T4/T5 precursors. Instead, neurons still develop in the mutant, but have neurite connectivity defects.

## Discussion

### Atonal and the insect visual circuit

Transcription factors of the Atonal family have previously been shown to act as proneural genes in many cellular contexts [28-30]. In the *Drosophila* visual system, Ato is the proneural gene for photoreceptor cells [18]. Interestingly, Ato also regulates indirectly the development of the optic lobe through the patterning of the retina and it is expressed during larval life in other populations of postmitotic optic lobe neurons, namely the dorsal cluster neurons and several clusters of neurons located ventrally in the brain [10]. In this work, we found that the development of T4/T5 neurons, essential components of the motion detection system, is also Ato dependent. Taken together these observations indicate that Ato underlies the patterning of the visual system at many different levels. We speculate that this may reflect a basal state of the invertebrate visual system in which an ancestral Ato-like proneural gene may have instructed the formation of all neural components in the visual system.

### A progenitor program regulates neurite guidance of neuronal progeny

In this work we identified a novel subset of *Drosophila* neural precursors where the proneural gene is required for the proper differentiation of the lineage. This is similar to previous observations in mammals. For example, mice mutant for the Neurogenin-2 proneural factor do not lack cortical neural stem cells but instead generate fewer neurons and these migrate and have neurite outgrowth defects [31,32], although in this case transient Ngn2 expression in postmitotic neurons has been observed [32].

The loss-of-function phenotype of Ato in IPC progenitors suggests two possible explanations. On the one hand, neural progenitors may initiate a transcriptional program prior to their division that is then required in their progeny for correct neurite targeting, implicating a cell autonomous function of Ato targets in the neurons. This may indicate that, as for the retina [33], the transcriptional program downstream of Ato in IPC progenitors regulates multiple aspects of lineage differentiation and likely the loss of several targets genes causes these defects. On the other hand, it is possible that the progenitors guide T4/T5 neurites during development and then the loss of function of Ato in the progenitors could affect, for instance, the expression of guidance molecules, indicating a non-autonomous mechanism. Future analysis of the Ato targetome in tissue-specific contexts will address these questions.

## Conclusions

Here we identified a proneural gene that is expressed in progenitors but is required for the guidance of their neuronal progeny. This suggests that transcriptional programs initiated specifically in progenitors are necessary for subsequent neuronal morphogenesis. These programs can act in two ways: either through the activation of a transcriptional cascade that survives cell division, or through direct guidance of neuronal processes by the progenitor cells themselves, through the expression or secretion of guidance factors.

## Methods

### Fly strains and genetic manipulation

Fly stocks were cultured on standard fly food. All experiments were performed in temperature-controlled incubators at 25°C or 28°C. The fly strains used were: CS10; *ato*^*1*^/TM6c; *ato*^*w*^*/*TM6c; *IPC(F1-R3)-Gal4*, *UAS-nEGFP* and *IPC(F1-R3)-Gal4*, *UAS-nEGFP;ato1*, *FRT82B/TM6b*, *IPC-EGFP* and *Ato-LexA*, *lexAop-TLNΔ-Cherry* for expression pattern and mutant analysis (in this case the leakage of the nEGFP from the nucleus allowed us to examine the neurites).

For the MARCM experiments, females with the genotype *hsFlp*, *UAS::CD8::GFP;;tub-Gal80*, *FRT82B/TM6c* were crossed with males *IPC(F1-R3)-Gal4*,*UAS-nEGFP; ato*^*1*^, *FRT82B/TM6c* or males *IPC(F1-R3)-Gal4*,*UAS-nEGFP; FRT82B/TM6c* (control) A 1-hr heat shock at 37°C was applied to the second instar larvae to induce mitotic recombination.

For the flybow experiments, *UAS-Flp*; *hs-mFlp5* stock was crossed with *UAS-Flybow2.0*; *IPC-Gal4* stock. A 1-hr heat shock at 37°C was applied to the third instar larvae (96 After egg lagging). The animals were dissected as late pupae (80% to 90%).

For dendritic-axon compartment analysis, flies tub < STOP > Gal4; *IPC-Gal4*/TM6c were crossed with the stock *UAS-Flp*; *UAS-DenMark*, *UAS-Syt::GFP* (*tub < STOP > Gal4* and UAS-Flp were used to prolong the expression of *IPC-Gal4*, which is not strong in the adult stage).

For the eye ablation experiments, *GMR-hid* flies were crossed with *IPC(F1-R3)-Gal4*, *UAS-nEGFP* flies.

### Antibody staining

For antibody staining, adult and larval brains were dissected in PBS and fixed in PBT 4% formaldehyde for 15 to 20 min. Fixed brains were washed three times for 15 to 20 min in PBT and incubated with the PAXDG buffer (PBT, 5% normal goat serum, 1% BSA, 0.3% deoxycholate) or PBT-BSA1% (for Ato antibodies), for 30 min to 1 hr at room temperature. Primary antibody incubation was done in PAXDG overnight at 4°C. Then the samples were washed three times with PBT and incubated with the appropriate secondary antibody in PAXDG for 2 to 4 hr, washed with PBT and mounted using the Vectashield mounting medium (Vector, Burlingame, CA, USA). The following antibodies were obtained from the Developmental Studies Hybridoma Bank (DSHB, Iowa city, IA, USA): rat anti-Elav (1:20), mouse anti-Bruchpilot (nc82 1:100), rat anti NCad (1:10) and mouse anti-Dlg (1:100). Other antibodies used were: sheep anti-Ato (1:1000), rabbit anti-Phospho-Histone-3 (Millipore, Billerica, MA, USA, 1:500), mouse anti-GFP 3E6 (Invitrogen, catalog number A11120, 1:250), rabbit anti-GFP (Invitrogen, Carlsbad, CA, USA, catalog number A11122, 1:500) and rabbit anti-DsRed (Clontech, Mountain View, CA, USA, catalog number 632496; 1:500). Secondary antibodies conjugated with Alexa 488, Alexa 555 and Alexa 647 were obtained from Invitrogen and used at 1:500. For the BrdU incorporation experiments, larval tissues were incubated in PBS with 75 μg/ml BrdU for 15 min, fixed and treated in 3 M HCl for 30 min before incubation with primary antibodies [34].

### Imaging

Imaging was performed using a Nikon A1-R confocal (Nikon, Tokyo, Japan) mounted on a Nikon Ti-2000 inverted microscope (Nikon) and equipped with 405-, 488-, 561- and 639-nm lasers from Melles Griotconfocal. Images were processed using the ImageJ software (National Institutes of Health, Bethesda, MD, USA). Figures were prepared using Adobe Photoshop (Adobe, San Jose, CA, USA). Cell clusters were quantified using the particle analysis tool of ImageJ.

### Statistics

Statistical analysis was performed using the Prism software (GraphPad Software Inc, La Jolla, CA, USA). A two-tailed *t*-test was used for two-group comparisons.

### IPC-GFP and IPC-Gal4

To generate *IPC-Gal4*, a sequence of 838 bp localized upstream of the *ato* locus (positions -5046 and -4209) was amplified by PCR using the primers:

Fw-Xba-I-IPC: TCTAGAGGATGGGATTAGGGATAAGG

Rv-Bgl-II-IPC: AGATCTGAATGGAGTTGGCCACAATGG

The amplified fragment was cloned in the Xba-I and Bgl-II restriction sites in the pPT-Gal4 vector.

For the *IPC-GFP* direct fusion, the IPC enhancer was cloned together with the Tata box of the pPT-Gal4 vector and an EGFP open reading frame in a modified pUAST vector. The UAS binding sites were deleted and an AttB site was added to allow PhiC31-mediated integration.

Transgenic flies were generated using standard methods.

## Abbreviations

APF: After pupal formation; AS: Achaete-Scute; Ato: Atonal; bHLH: basic helix-loop-helix; BrdU: bromodeoxyuridine; BSA: bovine serum albumin; Dlg: Disc Large; Elav: Embryonic lethal abnormal vision; GFP: Green fluorescent protein; GMR: Glass multiple reporter; EGFP: enhanced GFP IPC, inner proliferation center; L3: third instar larvae; La: lamina; Lo: lobula; Lop: lobula plate; MARCM: mosaic analysis with a repressible cell marker; Me: medulla; nEGFP: nuclear EGFP; OPC: outer proliferation center; PBS: phosphate-buffered saline; PBT: PBS plus 0,3% triton X100; PCR: polymerase chain reaction; Ph3: Phospho-Histone-H3; PNS: peripheral nervous system; Syt: Synaptotagmin; Tlp: translobula plate; UAS: Upstream Activating Sequence.

## Competing interests

The authors declare that they have no competing interests.

## Authors’ contributions

CO and CMC conceived and designed the study, collected and analyzed the data, wrote the manuscript and gave final approval for the manuscript. LJJN and NM collected and analyzed the data, and gave final approval for the manuscript. NDG carried out the cloning and provided technical assistance. BAH conceived and designed the study, participated in the coordination of the study, wrote the manuscript and gave final approval for the manuscript. All authors read and approved the final manuscript.

## Supplementary Material

Additional file 1: Figure S1 Relation between Ato promoter and the IPC enhancer. (A,B) Immunostaining in the L3 stage. (A) The LexA transcription factor, a knock-in in the *ato* locus, which drives TLNΔ-Cherry, is used to highlight the whole Ato lineage. There is co-localization with IPC-Gal4 at the border of the cluster (B) indicating that they coexist in some progenitors. Scale bar: A,B = 20 μm. Click here for file

Additional file 2: Figure S2 Neurons still develop in absence of Ato. (A-B') Immunostaining of wild-type and *ato*^*w *^L3 cells showing that in the mutants Ato^+^ progenitors (green) still give rise to Elav^+^ neurons (magenta). (A,A') Control animal bearing *IPC-Gal4*, *UAS-nEGFP* transgenes. (B,B') *ato*^*w *^mutant animals still express Elav in the cells originating from Ato^+^ precursors indicating that they are still neurons. **(C)** Quantification of the area of the IPC neuronal cluster in controls and *ato*^*w *^animals. There were no significant differences in the size of the cluster among the genotypes (two-tailed *t*-test, *P* = 0.8379). Scale bar: A-B' = 20 μm. Click here for file

Additional file 3: Figure S3 T4/T5 dendritic pattern is not disturbed when there is no retina. (A) Wild-type adult animal bearing *IPC-Gal4*, *UAS-nEGFP*. **(A')** Close-up showing the dendritic projections of the T4/T5 neurons. **(B)** Flies with no eyes because of expression of the pro-apoptotic gene *hid* using the GMR promoter. The dotted line in (B) marks the border between the lobula complex and the medulla. **(B')** Close-up showing the dendritic projections of the T4/T5 neurons in this condition. The neuropiles are smaller than the control but cell bodies and dendrites of the T4/T5 neurons have a wild-type appearance. Scale bars: A,B = 50 μm, A',B' = 25 μm. Click here for file
