# Supplementary figures and images for: Proper connectivity of Drosophila motion detector neurons requires Atonal function in progenitor cells
Source: Neural Dev. 2014 Feb 26;9:4. doi: 10.1186/1749-8104-9-4 (PMC3941608; doi:10.1186/1749-8104-9-4)

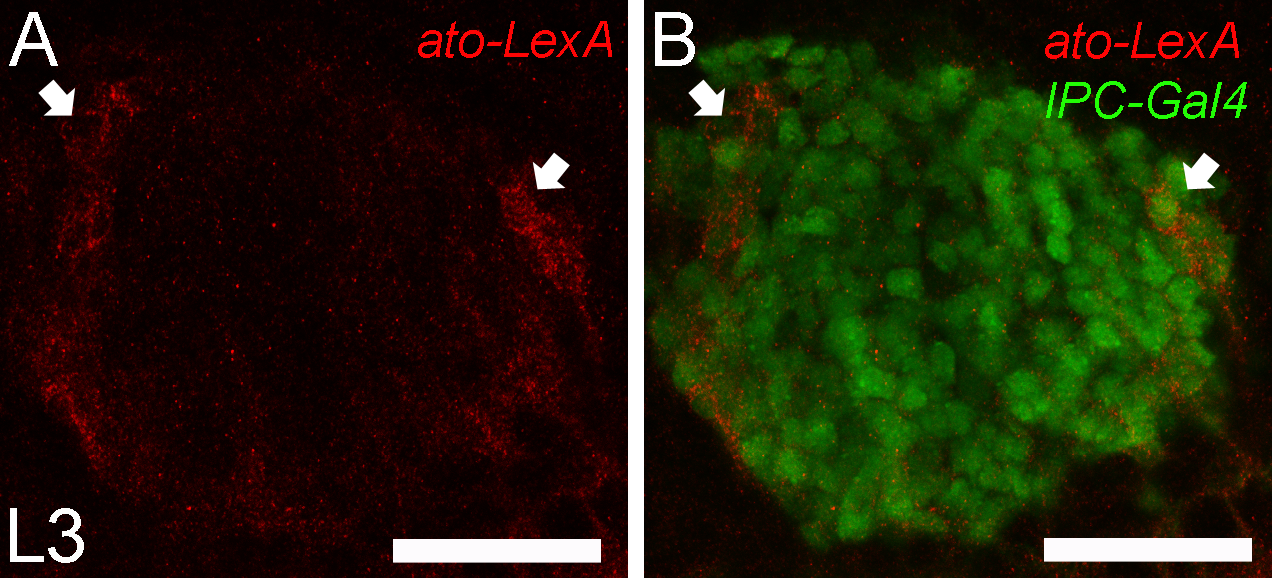

Supplement: Additional file 1: Figure S1 — Relation between Ato promoter and the IPC enhancer. (A,B) Immunostaining in the L3 stage. (A) The LexA transcription factor, a knock-in in the ato locus, which drives TLNΔ-Cherry, is used to highlight the whole Ato lineage. There is co-localization with IPC-Gal4 at the border of the cluster (B) indicating that they coexist in some progenitors. Scale bar: A,B = 20 μm. [file 1749-8104-9-4-S1.tif]

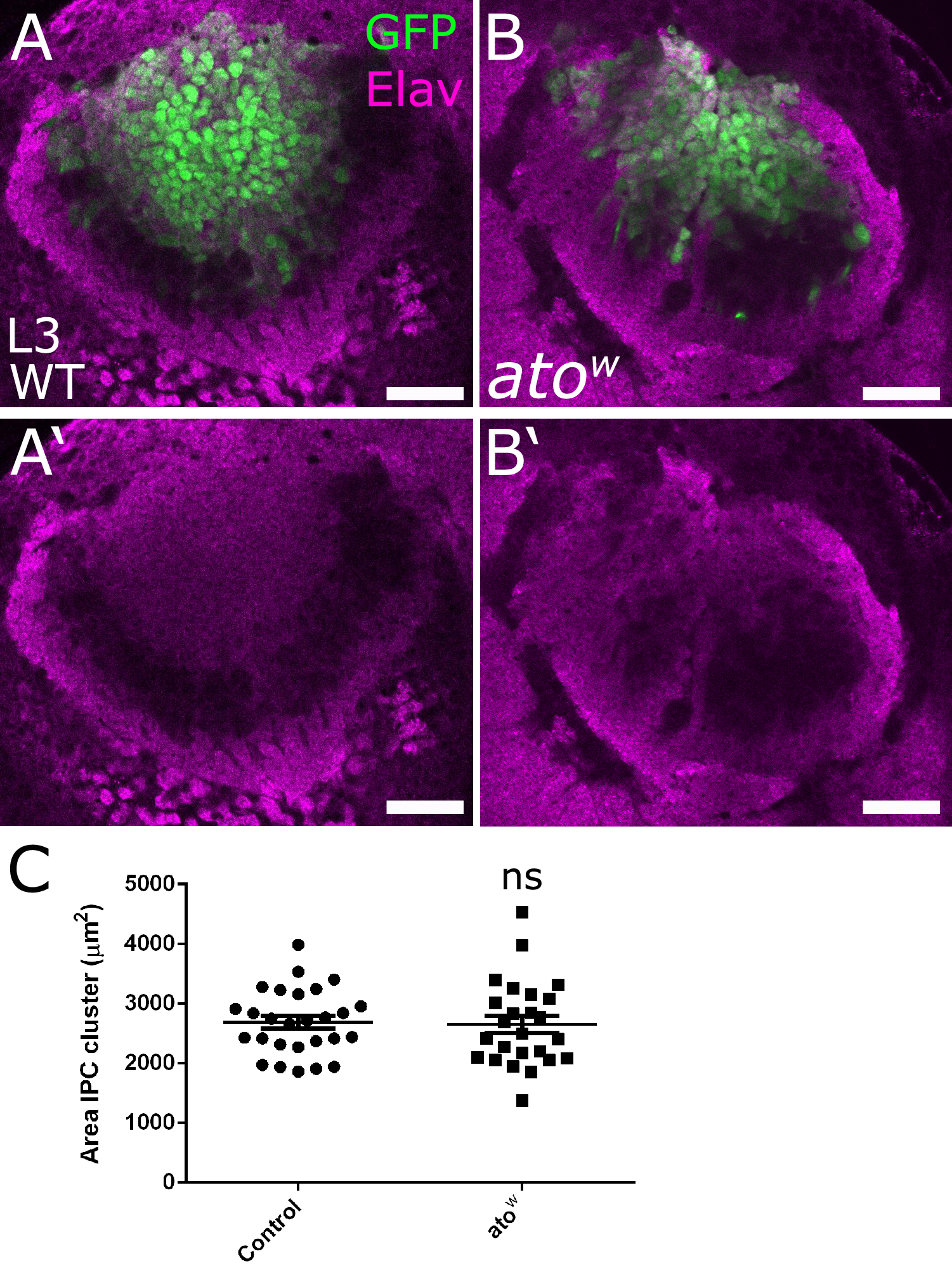

Supplement: Additional file 2: Figure S2 — Neurons still develop in absence of Ato. (A-B') Immunostaining of wild-type and ato w L3 cells showing that in the mutants Ato+ progenitors (green) still give rise to Elav+ neurons (magenta). (A,A') Control animal bearing IPC-Gal4, UAS-nEGFP transgenes. (B,B') ato w mutant animals still express Elav in the cells originating from Ato+ precursors indicating that they are still neurons. (C) Quantification of the area of the IPC neuronal cluster in controls and ato w animals. There were no significant differences in the size of the cluster among the genotypes (two-tailed t-test, P = 0.8379). Scale bar: A-B' = 20 μm. [file 1749-8104-9-4-S2.tif]

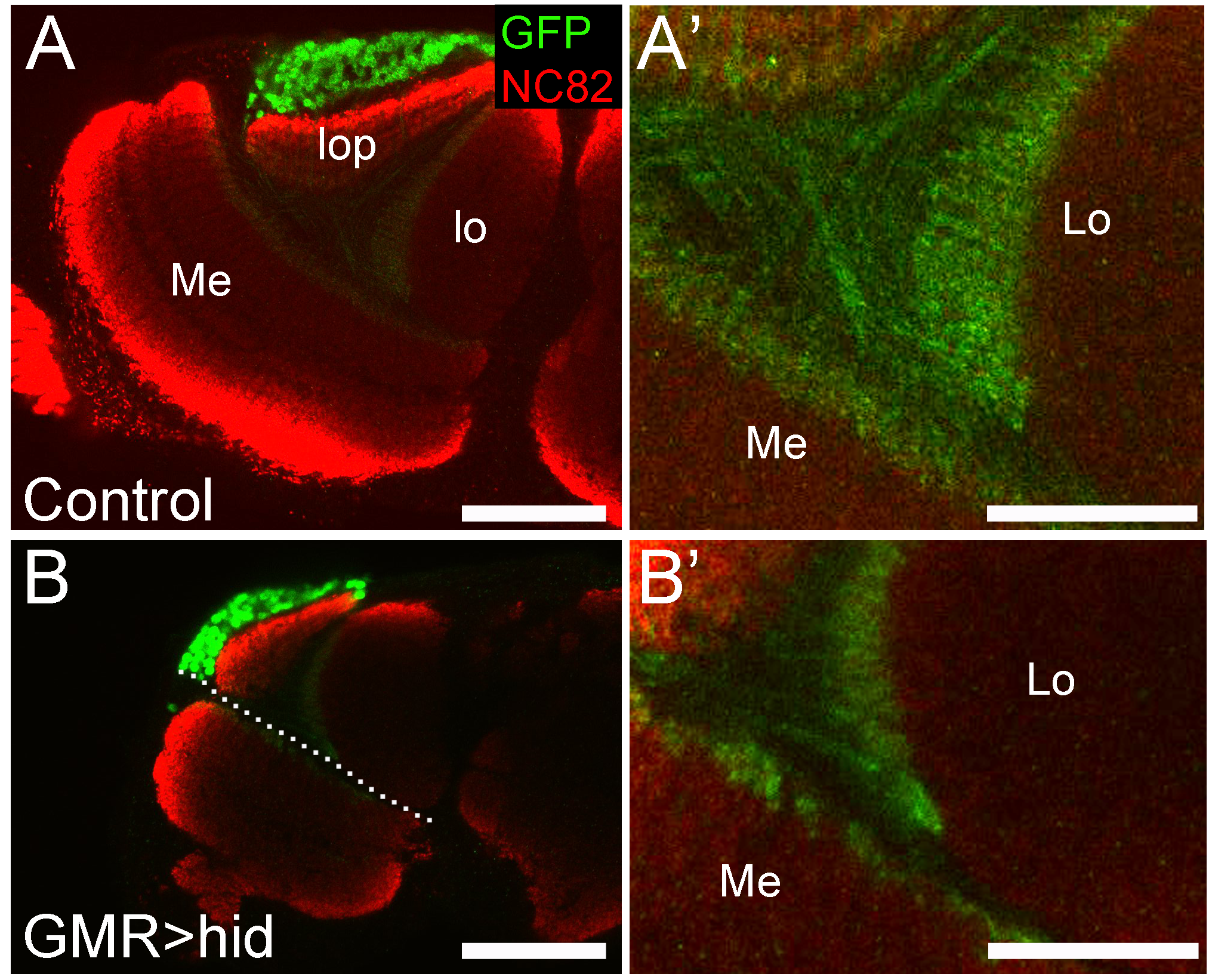

Supplement: Additional file 3: Figure S3 — T4/T5 dendritic pattern is not disturbed when there is no retina. (A) Wild-type adult animal bearing IPC-Gal4, UAS-nEGFP. (A') Close-up showing the dendritic projections of the T4/T5 neurons. (B) Flies with no eyes because of expression of the pro-apoptotic gene hid using the GMR promoter. The dotted line in (B) marks the border between the lobula complex and the medulla. (B') Close-up showing the dendritic projections of the T4/T5 neurons in this condition. The neuropiles are smaller than the control but cell bodies and dendrites of the T4/T5 neurons have a wild-type appearance. Scale bars: A,B = 50 μm, A',B' = 25 μm. [file 1749-8104-9-4-S3.tif]
